# Case Report: CASPR2-associated autoimmune encephalitis with NF155 Antibody-positive autoimmune nodopathies: a rare case with hyponatremia onset

**DOI:** 10.3389/fimmu.2025.1519878

**Published:** 2025-03-14

**Authors:** Wen-Ya Wang, Jing-Ying Huang, Ying Xue, An-Ran Zhang, Ruo-Yi Guo, Zhen Jia, Ya-Fei Sun, Bin Li

**Affiliations:** ^1^ Department of Neurology, The Second Hospital of Hebei Medical University, Shijiazhuang, Hebei, China; ^2^ Key Laboratory of Clinical Neurology (Hebei Medical University), Ministry of Education, Shijiazhuang, Hebei, China; ^3^ Neurological Laboratory of Hebei Province, Shijiazhuang, China

**Keywords:** CASPR2, NF155, autoimmune encephalitis, autoimmune nodopathies, hyponatremia

## Abstract

**Objectives:**

This case report aims to highlight the atypical presentation and management of a patient diagnosed with CASPR2-associated autoimmune encephalitis and NF155 antibody-positive autoimmune nodopathies (AN), initially presenting with limb weakness and hyponatremia.

**Methods:**

The patient was identified through clinical evaluation and diagnostic testing including serum and cerebrospinal fluid analysis, neuroimaging, and nerve conduction studies.

**Results:**

The patient exhibited limb weakness, hyponatremia, cognitive abnormalities, and peripheral nerve involvement. Diagnostic testing revealed CASPR2 and NF155 antibody are positive. Therapeutic interventions included corticosteroids, plasma exchange, and intravenous immunoglobulin therapy, followed by B-cell depletion therapy. Treatment led to improvement in walking function and normalization of antibodies.

**Discussion:**

This case report contributes to the literature by documenting a rare co-occurrence of CASPR2-associated autoimmune encephalitis and NF155 antibody-positive AN, with a unique presentation of hyponatremia. The findings underscore the importance of considering autoimmune etiologies in patients presenting with hyponatremia and neurological symptoms. Moreover, the favorable response to B-cell depletion therapy suggests a potential treatment option for similar cases. The main take away is the need for heightened clinical suspicion and comprehensive diagnostic evaluation in patients with complex neurological presentations, to facilitate timely diagnosis and appropriate management.

## Introduction

Autoimmune nodopathies (AN) are now recognized as distinct from traditional inflammatory demyelinating neuropathies, as patients with this condition exhibit characteristic paranodal axo-glial detachment (1). Both CASPR2-associated autoimmune encephalitis and AN are classified under IgG4 autoimmune diseases (IgG4-AID), characterized by the presence of pathogenic IgG4 antibodies. These antibodies primarily target antigens in various organ systems, including the central and peripheral nervous system (2). In this case report, we present a unique instance of dual antibody positivity affecting both the central and peripheral nervous systems.

## Case history

A 70-year-old male presented with weakness in all four limbs after defecation, particularly prominent in the lower limbs, which rendered him unable to stand up independently. He also reported mild numbness in his limbs.The electrolyte showed Na^+^:107 mmol/L and after correcting hyponatremia, he could walk normally. However, there was still numbness in lower limbs and hands. After that, he had symptoms of sweating.

One month before hospitalization, the weakness of lower limbs reappeared, which was gradually aggravated. The electrolyte showed Na+:110 mmol/L, and the symptom improved again after sodium supplementation.

Half a month before hospitalization, he went to the endocrinology department for the recurrence of the above symptoms. 24-hour urine volume: 1.62 L, 24-hour urine sodium: 251.42 mmol/24h. Blood electrolyte: sodium: 117.9 mmol/L. 24h urinary protein: 3.68 g/24h. Urine osmotic pressure: 499 mOsm/(kg.H_2_O), blood osmotic pressure: 266 mOsm/(kg.H_2_O). The patient underwent comprehensive endocrine evaluations, including thyroid function, plasma cortisol levels, adrenocorticotropic hormone (ACTH), insulin-like growth factor-1 (IGF-1), and serum C-peptide measurements, all of which showed no significant abnormalities.No abnormal signals were found in head MRI, cervical MRI and thoracic MRI. PET-CT was normal. Finally, he was diagnosed with syndrome of inappropriate antidiuretic hormone (SIADH). After treatment with tolvaptan and other treatments, the blood sodium was close to normal, but the symptoms did not improve. And then he transferred to our department for further treatment.

He has many diseases, such as hypertension, diabetes, coronary heart disease and paroxysmal atrial fibrillation. We could see that there was intentional tremor in his hands and muscle twitching in his legs. Muscle strength of both upper limbs was V^-^ level, left lower limb was IV level, right lower limb was III level. Tendon reflex of both upper limbs was decreased, and both lower limbs were not led out. In four limbs, the distal superficial sensation decreased, and the left lower limb position sensation decreased. Both finger nose tests and the left lower limb heel-knee-shin test were not accurate.

No obvious abnormality was found in serum free light chain, urine protein electrophoresis and immunofixation electrophoresis. Cerebrospinal fluid pressure was normal, and the protein was increased, which was 1.22 g/L. White blood cell count was normal. Cytological showed that there were 12 lymphocytes, 3 monocytes and scattered red blood cells. MMSE: 24, MoCA: 13, suggesting he had cognitive abnormality. EEG showed that there was slow wave of background activity. Electrophysiological studies revealed slowed motor and sensory conduction velocities, reduced amplitudes, prolonged F-wave latencies, and the presence of a few afterpotentials (**Table 1**). Electromyography indicated neurogenic damage in the bilateral tibialis anterior, gastrocnemius, abductor pollicis brevis, and the first dorsal interosseous muscles.Antibodies to autoimmune encephalitis in serum and CSF were tested by cell-based assay. CASPR2 antibody was positive in serum (titer 1: 1000) and cerebrospinal fluid (titer 1: 100). Serum anti-NF155 antibody positive: 1: 100. The patient was diagnosed as CASPR2-associated autoimmune encephalitis and AN. Considering that the patient was complicated with various medical diseases, we gave 80mg methylprednisolone. Besides, plasma exchange was performed for 3 times. The patient’s muscle strength did not recover well, but the sensory system improved. Intravenous immunoglobulin therapy (IVIG) was applied in sequence. The dosage was 0.4 g/kg, 25 g each time, and it was used for 3 days.

**Table 1:** The detailed electrophysiologic data of the patient.

Detailed electrophysiologic data (Motor/F-wave)				
	DML, ms	CMAP, mV	CV, m/s	F-DML, ms
Median nerve (L/R)	12.0/11.7	1.4↓/3.5↓	30.4↓/31.1↓	49.1↑/49.4↑
Ulnar nerve (L/R)	11.8/11.8	4.2/3.4	35.7↓/41.7↓	49.4↑/49.3↑
Peroneal nerve (L/R)	18.7/17.8	1.0↓/0.9↓	23.5↓/27.7↓	89.4↑/88.8↑
Tibial nerve (L/R)	19.8/20.6	0.6↓/0.4↓	27.9↓/26.4↓	89.4↑/90.3↑
Detailed electrophysiologic data (Sensory)				
	DML, ms	CMAP, mV	CV, m/s	
Median nerve (L/R)	5.1/4.9	1.9↓/1.8↓	27.6↓/28.8↓	
Ulnar nerve (L/R)	4.1/4.0	2.9↓/1.1↓	29.4↓/30.3↓	
Sural nerve (L/R)	3.0/2.9	6.4/6.2	42.8/44.4	

DML, distal motor latency; CMAP, compound muscle action potential; CV, conduction velocity; F-DML, F-wave-distal motor latency; L, left; R, right.

↓, descend; ↑, rise.

**Figure 1:**
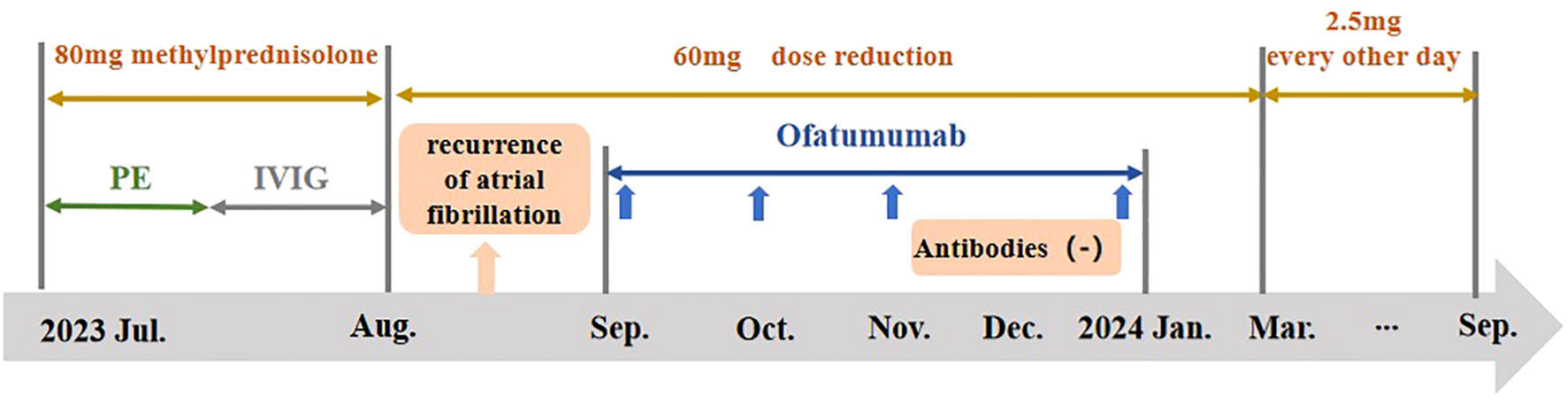
The treatment in the case. The patient’s treatment consists of two phases: the acute phase and the remission phase. The acute phase treatment includes PE, IVIG and 80mg of methylprednisolone. The remission phase treatment mainly involves subcutaneous injections of Ofatumumab. In August, the patient was hospitalized due to a recurrence of atrial fibrillation. The blue arrow shows each application of Ofatumumab. CASPR2 antibody and NF155 antibody turned negative in December 2023. By September 2024, the methylprednisolone was tapered off and discontinued. IVIG, Intravenous immunoglobulin therapy; PE, plasma exchange.

After discharge, he took 60 mg of prednisone acetate every day, reducing 5 mg every week. A month later, the patient was hospitalized because of the recurrence of atrial fibrillation. In terms of walking, he was limited to walking a few steps with help. Taking into account the potential side effects of hormonal treatments and the challenges of physical recovery, we incorporated Ofatumumab into the treatment regimen. He took one injection every month. After 3 months of regular application, the antibody reexamination showed that CASPR2 antibody and NF155 antibody had turned negative. During the latest follow-up(September 2024), the patient could walk 500m without the aid and discountinued the hormone therapy. The amplitude and frequency of intentional tremor of both hands are greatly reduced compared with before. Figure displays the chronological sequence of the patient’s clinical interventions (**Figure 1**).

## Discussion

In this study, we describe a patient who started with weakness of limbs and hyponatremia, and he was finally diagnosed as CASPR2-associated autoimmune encephalitis with NF155 antibody positive AN. So far, no cases of both positive cases have been reported. CASPR2-associated autoimmune encephalitis typically presents with symptoms such as seizures, psychiatric and behavioral abnormalities, and in some cases, peripheral nerve hyperexcitability symptoms like myokymia and myotonia, which may be accompanied by neuropathic pain. However, reports of hyponatremia are rare. There is also no report of hyponatremia in anti-NF155 AN. The patient started with hyponatremia, and the remission and aggravation of the early symptoms seemed to be related to the blood sodium level. This is what makes the report unusual. The common cause of hyponatremia in clinic is SIADH, accounting for about 31%. Severe craniocerebral injury, severe intracranial infection, acute cerebrovascular disease and so on can all cause SIADH. In autoimmune encephalitis, LGI1 encephalitis is easy to be complicated with SIADH (3). Irani SR et al. found that LGI1 antibody can bind to the cell bodies of orexin neurons in the outboard hypothalamus and ADH neurons in the inboard hypothalamus. CASPR2 is mainly expressed in parasynaptic of white matter and nerve fibers, and is not co-located with orexin or ADH neurons (4). This suggests that another mechanism may be needed to explain the cause of hyponatremia caused by CASPR2 antibody.

Through the studies of various molecules and their interactions, the pathogenic mechanism of antibodies has been gradually clarified. In molecular structure, CASPR2 and NF155 are located near node. CASPR2 is located in juxtaparanode and NF155 is located in paranode. NF155 is connected with CNTN1 and CASPR1 on the axonal cell membrane to form a transverse band. CASPR2 interacts with contact protein 2, which promotes the aggregation of Kv1 potassium channels in the juxtaparanode. At the amino acid level, CASPR2 is 45% identical to CASPR1, exhibiting many structural similarities (5). Therefore, our patients are both positive for CASPR2 and NF155 antibodies. Whether this is related to structural similarity and close location needs further study.

Although further IgG subclass determination was not performed in this particular case, studies have demonstrated that CASPR2 autoantibodies are predominantly of the IgG4 subtype (6, 7). Similarly, in cases of CIDP with anti-NF155 antibodies, IgG4 is also the predominant subclass (8). Currently, it is hypothesized that IgG4 plays a direct pathogenic role in IgG4-AID by impeding protein-protein interactions (9). However, the response of most patients to immunoglobulin therapy is not readily apparent, largely owing to the distinctive nature of IgG4, a trait shared by our patient. IgG4 antibodies are primarily generated by short-lived plasmablasts/plasma cells (10). A study has suggested that rituximab serves as an effective therapy for anti-NF155 AN (11). Remarkably, our patient exhibited a favorable response to B-cell depletion therapy. This clinical improvement, coupled with the normalization of blood sodium levels and the disappearance of antibodies following Ofatumumab administration, underscores the efficacy of this treatment approach.

In a word, our report describes the phenomenon of the comorbidity of CASPR2-associated autoimmune encephalitis and anti-NF155 AN for the first time. It also broadens the range of double positive antibody and the clinical features of CASPR2 encephalitis, such as hyponatremia. When patients start with hyponatremia, besides LGI1 encephalitis, the possibility of this type of encephalitis should also be considered.

## Data Availability

The raw data supporting the conclusions of this article will be made available by the authors, without undue reservation.

## References

[1] Koike H Kadoya M Kaida KI Ikeda S Kawagashira Y Iijima M. Paranodal dissection in chronic inflammatory demyelinating polyneuropathy with anti-neurofascin-155 and anti-contactin-1 antibodies. J Neurol Neurosur Ps. (2017) 88:465–73. doi: 10.1136/jnnp-2016-314895 28073817

[2] Koneczny I. Update on igG4-mediated autoimmune diseases: new insights and new family members. Autoimmun Rev. (2020) 102646. doi: 10.1016/j.autrev.2020.102646 32801046

[3] Lai M Huijbers MG Lancaster E Graus F Bataller L Balice-Gordon R. Investigation of LGI1 as the antigen in limbic encephalitis previously attributed to potassium channels: a case series. Lancet Neurol. (2010) 9:776–85. doi: 10.1016/S1474-4422(10)70137-X PMC3086669 20580615

[4] Irani SR Pettingill P Kleopa KA Schiza N Waters P Mazia C. Morvan syndrome: clinical and serological observations in 29 cases. Ann Neurol. (2012) 72:241–55. doi: 10.1002/ana.23577 22473710

[5] Zou Y Zhang WF Liu HY Li X Zhang X Ma XF. Structure and function of the contactin-associated protein family in myelinated axons and their relationship with nerve diseases. Neural Regener Res. (2017) 12:1551–8. doi: 10.4103/1673-5374.215268 PMC5649478 29090003

[6] van Sonderen A Ariño H Petit-Pedrol M Leypoldt F Körtvélyessy P Wandinger KP. The clinical spectrum of Caspr2 antibody-associated disease. Neurology. (2016) 87:521–8. doi: 10.1212/WNL.0000000000002917 PMC4970662 27371488

[7] Patterson KR Dalmau J Lancaster E. Mechanisms of Caspr2 antibodies in autoimmune encephalitis and neuromyotonia. Ann Neurol. (2018) 83:40–51. doi: 10.1002/ana.25120 29244234 PMC5876120

[8] Kira JI. Anti-neurofascin 155 antibody-positive chronic inflammatory demyelinating polyneuropathy/combined central and peripheral demyelination: strategies for diagnosis and treatment based on the disease mechanism. Front Neurol. (2021) 12:665136. doi: 10.3389/fneur.2021.665136 34177770 PMC8222570

[9] oneczny I Tzartos J Mané-Damas M Yilmaz V Huijbers MG Lazaridis K. IgG4 autoantibodies in organ-specific autoimmunopathies: reviewing class switching, antibody-producing cells, and specific immunotherapies. Front Immunol. (2022) 13:834342. doi: 10.3389/fimmu.2022.834342 35401530 PMC8986991

[10] Unger PA Lighaam LC Vermeulen E Kruithof S Makuch M Culver EL. Divergent chemokine receptor expression and the consequence for human IgG4 B cell responses. Eur J Immunol. (2020) 50:1113–25. doi: 10.1002/eji.201948454 32289181

[11] Martín-Aguilar L Lleixà C Pascual-Goñi E Caballero-Ávila M Martínez-Martínez L Díaz-Manera J. Clinical and laboratory features in anti-NF155 autoimmune nodopathy. Neurol Neuroimmunol Neuroinflamm. (2021) 9:e1098. doi: 10.1212/NXI.0000000000001098 34728497 PMC8564865

